# Impact of white matter hyperintensities on the prognosis of cryptogenic stroke patients

**DOI:** 10.1371/journal.pone.0196014

**Published:** 2018-04-27

**Authors:** Seong Ho Jeong, Sung Soo Ahn, Minyoul Baik, Ki Hoon Kim, JoonSang Yoo, Kyoungsub Kim, Hye Sun Lee, Jimin Ha, Young Dae Kim, Ji Hoe Heo, Hyo Suk Nam

**Affiliations:** 1 Department of Neurology, Yonsei University College of Medicine, Seoul, Korea; 2 Department of Radiology, Yonsei University College of Medicine, Seoul, Korea; 3 Department of Neurology, Keimyung University School of Medicine, Daegu, Korea; 4 Department of Biostatistics, Yonsei University College of Medicine, Seoul, Korea; 5 Brain Korea 21 Plus Project for Medical Science, Yonsei University, Seoul, Korea; Universitair Medisch Centrum Utrecht, NETHERLANDS

## Abstract

**Background:**

To our knowledge, little is known regarding whether white matter hyperintensities (WMH) affect the prognosis of cryptogenic stroke (CS) patients. Understanding this association may be helpful with expecting the prognosis of CS patients.

**Methods:**

This retrospective observational study enrolled consecutive CS patients who underwent brain MRI and comprehensive cardiac evaluation. Severe WMH was defined as Fazekas’ score ≥3. We defined poor functional outcome as modified Rankin Scale score ≥3 at 3 months. Long-term mortality and causes of death were identified using national death certificates and assessed by Kaplan-Meier method and regression analysis model.

**Results:**

Among 2732 patients with first-ever ischemic stroke, 599 (21.9%) patients were classified as having CS. After exclusions, 235 patients were enrolled and followed up for a median of 7.7 years (IQR, 6.7–9.0). Severe WMH were found in 81 (34.5%) patients. After adjustments, severe WMH were an independent predictor for poor functional outcomes at 3 months (OR 5.25, 95% CI, 2.07–13.31). Subgroup analysis showed that severe WMH were an independent predictor for long-term mortality only in younger patients (age < 65) (HR 3.11, 95% CI, 1.29–7.50), but not in older patients (HR 1.19, 95% CI, 0.63–2.23).

**Conclusions:**

Severe WMH were independently associated with short-term functional outcomes in CS patients and independently associated with long-term mortality in younger CS patients. Grading WMH is of value in predicting prognosis of CS patients with young age.

## Introduction

The etiology of cryptogenic stroke (CS) is unknown in about 20 to 25% of stroke patients despite extensive evaluation. The occurrence of CS is more common in young stroke patients than in older patients [1]. According to stroke outcome studies, 14% to 30% of CS patients experience recurrent stroke and 35.8% to 41% have poor short-term outcomes [2, 3]. Though age, vascular risk factors, and infarct volume are sharing prognostic factors of all stroke subtypes [4–6], specific prognostic factors associated with CS need to be investigated.

White matter hyperintensities (WMH) are small vessel diseases defined as patchy or confluent lesions in periventricular or subcortical areas. These lesions are usually identified by high signal intensity in T2-weighted or fluid-attenuated inversion recovery (FLAIR) MRI [7]. WMH is strongly linked to lacunar infarction as they share common small artery pathologies [8]. WMH is an important imaging biomarker of brain frailty [9]. Long-term prognoses of patients with WMH are typically poor. Poor prognoses associated with WMH are reported not only in the general patient population [10, 11], but also in stroke patients [12, 13]. Specifically, There were several reports that WMH burden influenced prognosis in stroke subtypes of lacunar infarctions and large artery atherosclerosis [14, 15].

Because WMH is a chronic process and CS is more frequent in younger patients, the impacts of these conditions on mortality are difficult to determine through short-term follow-up periods. In addition, since elderly stroke patients are more likely to die than younger patients and they tend to have multiple comorbidities, it might be needed to test long-term prognosis according to age stratification. However, little is known about the long-term prognosis of CS patients with higher WMH burden according to age. In this regard, we hypothesized and investigated that WMH burden is a surrogate imaging marker of long-term outcome especially in younger CS patients.

## Methods

### Patients and evaluation

This was a retrospective observational study for CS patients who were prospectively enrolled in the single center stroke registry [16]. During admission, all patients were thoroughly investigated for medical history, clinical manifestations, and vascular risk factors. Every patient was evaluated with 12-lead electrocardiography, chest x- ray, lipid profiles, and standard blood tests. All registered patients underwent brain imaging studies including brain computed tomography (CT) and/or MRI. Angiographic studies using CT angiography, magnetic resonance angiography, or digital subtraction angiography were included in standard evaluations. Additional blood tests for coagulopathy or prothrombotic conditions were performed in patients younger than 45 years old. Transesophageal echocardiography was included in the standard evaluation, except in patients with decreased consciousness, impending brain herniation, poor systemic condition, inability to accept an esophageal transducer because of swallowing difficulty or tracheal intubation, or lack of informed consent [17]. Transthoracic echocardiography, heart CT, and Holter monitoring were also performed in selected patients [18]. Most patients were admitted to the stroke unit and monitored continuously with EKG during their stays. For this study, we included consecutive patients admitted from Jan 2001 to June 2007. When a patient was admitted more than twice because of recurrent strokes, only data for the first admission were used for this study.

### Risk factor definitions

Hypertension was defined as resting systolic blood pressure ≥140 mm Hg or diastolic blood pressure ≥90 mm Hg after repeated measurements during hospitalization or currently taking antihypertensive medication. Diabetes mellitus was defined as fasting plasma glucose values ≥7 mmol/L or taking an oral hypoglycemic agent or insulin. Hyperlipidemia was diagnosed as a fasting serum total cholesterol level ≥6.2 mmol/L, low-density lipoprotein cholesterol ≥4.1 mmol/L, or currently taking a lipid-lowering drug after a hyperlipidemia diagnosis. A current smoker was defined as an individual who smoked at the time of stroke or had quit smoking 1 year prior to treatment [19].

### Stroke subtype classification

Stroke classifications were determined during weekly conferences based on the consensus of stroke neurologists. Data including clinical information, risk factors, imaging study findings, laboratory analyses, and other special evaluations were collected. Along with these data, prognosis during hospitalization and long-term outcomes were also determined. Data were entered into a web-based registry. Stroke subtypes were identified according to the Trial of ORG 10172 in Acute Stroke Treatment (TOAST) classification [20]. We defined CS as strokes of undetermined etiology attributable to negative evaluation despite extensive work-up.

### Neuroimaging protocol

MRI examinations were performed on either a 1.5T (Signa Horizon 1.5T 85, GE Medical System, Milwaukee, Wis; or Intera 1.5T, Philips Medical Systems, Best, Netherlands) or a 3.0T MRI system (Achieva 3.0T, Philips Medical Systems, Best, the Netherlands). Patients underwent MRI within 3 days after admission according to the following parameters. For diffusion-weighted imaging: repetition time (TR)/echo time (TE) = 2,600–6,500/42–70 milliseconds, 2-mm interslice gap, field of view (FOV) = 230 X 230 mm, slice thickness = 5 mm, 6 different diffusion gradient directions (x, y, z, xy, yz, zx), and 2 b values (0, 1,000); for fluid-attenuated inversion recovery: TR/TE = 9,000/120 milliseconds, pixel spacing = 0.449 mm/0.449 mm, FOV = 230 X 230 mm, and slice thickness = 5 mm.

### Image review and analysis

WMH were assessed on FLAIR images in the supra tentorium according to the Standards for Reporting Vascular Changes on Neuroimaging criteria [21]. WMH was graded according to the Fazekas scale, which has been widely used for rating WMH [22]. We graded WMH in the hemisphere contralateral to acute stroke because WMH may overlap with the acute infarction area. When a patient had bilateral small infarctions, the less-involved hemisphere was measured [23]. Using visual assessment of FLAIR images, we further categorized WMH into periventricular and deep WMH. Periventricular WMH indicated WMH exclusively located around the lateral ventricles (0: absent, 1: caps or pencil lining, 2: smooth halo, 3: irregular periventricular WMH extending into deep white matter). Deep WMH denoted WMH in the centrum semiovale and the corona radiata (0: absent, 1: punctate foci, 2: beginning confluence of foci, 3: large confluent areas). Total WMH score was calculated by summing the scores for periventricular WMH and deep WMH, and ranged between 0 and 6. WMH degree was trichotomized into no (Fazekas score = 0), mild (Fazekas score = 1 to 2), and severe (Fazekas score = 3 to 6). Two investigators blinded to clinical information reviewed the MRI and rated WMH degree. Inter-rater reliability between investigators was excellent (κ values = 0.842 for periventricular WMH and 0.798 for deep WMH).

According to the TOAST classification, CS patients should not have greater than 50% atherosclerosis in the relevant artery. Therefore, we defined atherosclerotic lesions of less than 50% as mild atherosclerosis. We classified the study cohort into four groups based on the location of arterial stenosis: (1) intracranial (IC), which included patients who had stenotic lesions in the IC internal carotid arteries, the IC vertebral arteries, the basilar artery, proximal portion of the middle cerebral arteries, the anterior cerebral arteries and the posterior cerebral arteries without significant stenosis in extracranial (EC) arteries; (2) EC, which included patients with stenotic lesions in EC arteries, such as the proximal internal carotid arteries or the EC vertebral arteries; (3) mixed, which included patients with stenotic lesions in both IC and EC arteries; and (4) non-stenosis [24].

### Follow-up and outcome measures

Surviving patients were followed in outpatient clinic or by a structured telephone interview at 3 months and every year after discharge. Short-term functional outcomes at 3 months were determined based on the modified Rankin Scale. Poor outcome was defined as a modified Rankin Scale (mRS) score ≥3. Pre-stroke mRS score was not measured. Deaths among subjects from January 2001 to December 31, 2013 were confirmed by matching the information in the death records and identification numbers assigned to the subjects at birth [25]. We obtained data for the date and causes of death from the Korean National Statistical Office, which were identified based on death certificates. The causes of death were coded according to the ICD-10. Cardiovascular death included fatal stroke (I60–64) and fatal ischemic heart disease caused by myocardial infarction (I21–23, I46).

### Standard protocol approvals, registrations, and patient consents

The institutional review board of Severance Hospital, Yonsei University Health System, approved this study and waived the patients’ informed consent because of a retrospective design and observational nature of this study.

### Statistical analysis

SPSS for Windows (version 23, SPSS, Chicago, IL, USA) was used for statistical analysis. The Pearson χ2 test was used to compare frequencies. For continuous variables, normality was examined using the Kolmogorov-Smirnov test. If the data did not deviate from a normal distribution, the mean and standard deviation were calculated and independent sample t-tests were used for comparisons. For data that were not normally distributed, we reported descriptive statistics as the median and interquartile range (IQR) and compared them using the Mann-Whitney U test. Logistic regression analysis was used to identify factors associated with severe WMH and poor outcomes at 3 months. Survival curves were generated according to the Kaplan-Meier method and compared using the log-rank test. The Cox proportional hazard regression analysis was performed to calculate crude and adjusted hazard ratios with 95% CI. The interaction term (age*severe WMH) was also tested in the regression model. Because age is a major determinant for WMH, subgroup analysis was performed by dichotomizing using the median age (64 years). Younger patients were defined as those <65 years old and older patients were ≥65 years old. Stroke severity was stratified on the basis of NIHSS as follows: mild (0–5), moderate (6–14) and severe (≥15) [26]. All parameters that were significant in the univariable analysis (p<0.1) and possible confounding factors including age and gender were included in the multivariable model. Statistical significance was set at p <0.05.

## Results

### Patient enrollments and stroke etiology evaluation

**Fig 1:**
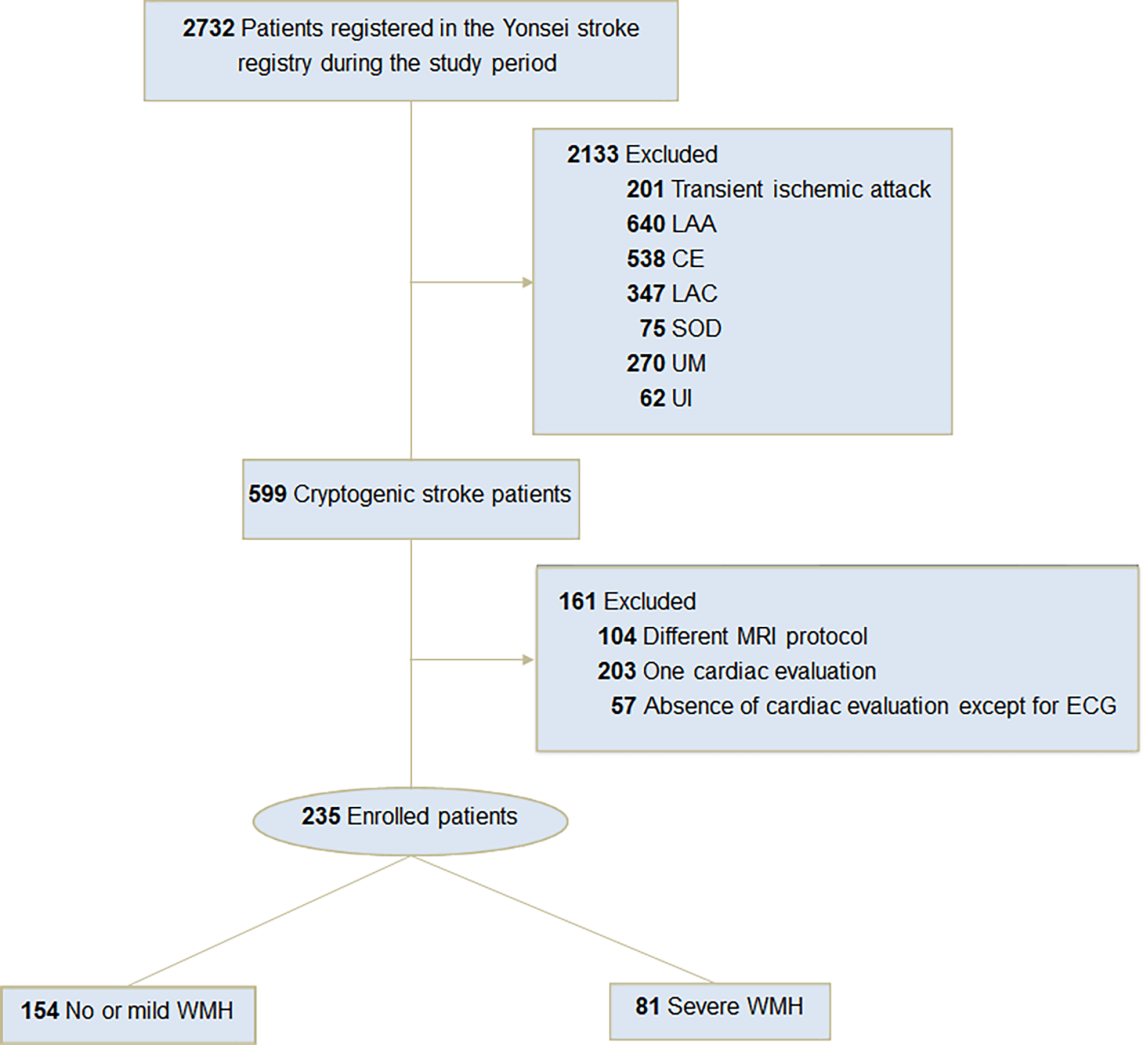
Patient flow chart. WMH indicates white matter hyperintensities; LAA, large artery atherosclerosis; CE, cardioembolism; LAC, lacune; SOD, stroke of other determined etiology; UM, stroke of undetermined etiology because of multiple causes; UI, stroke of undetermined etiology because of incomplete evaluation.

Total of 2732 patients were registered in the stroke registry during the study period. After excluding 201 patients with TIA and 1932 patients with non-cryptogenic stroke, a total of 599 patients (21.9%) were classified as having CS. Among them, 104 patients (17.4%) who underwent different protocols and 260 (43.4%) patients who did not undergo comprehensive cardiac evaluations were excluded. Demographic characteristics of excluded CS patients were not different except for age and the proportion of female (described in S1 Table). Finally, 235 patients were enrolled in the study (Fig 1). Angiographic evaluations of supra-aortic trunks were performed for all patients.

### Demographic data and factors associated with severe white matter hyperintensities

The median age was 63 (IQR, 54–69 years) and 32.3% were woman. Among the enrolled patients, 214 patients (91.1%) had WMH and 21 patients (8.9%) did not have WMH. Degree of WMH was severe in 81 patients (34.5%) and mild in 236 patients (56.6%). Degree of WMH in the periventricular area was correlated with that of deep white matter (correlation coefficient = 0.773, p<0.001). When comparing patients with no or mild WMH, those with severe WMH were older, more likely to be woman, less likely to be current smoker, more frequently had histories of hypertension, and cerebral artery atherosclerosis. Mild cerebral artery atherosclerosis (arterial stenosis <50%) was found in 55.3% of patients. Cerebral artery atherosclerosis is correlated with degree of WMH (p <0.001), especially in IC atherosclerosis (Table 1). In univariable analysis, old age, woman, hypertension, smoking, and intracranial or combined atherosclerosis in both IC and EC were associated with severe WMH. In multivariable logistic regression analysis, old age, hypertension, and IC or combined atherosclerosis in both IC and EC were independently associated with severe WMH (described in S2 Table).

**Table 1:** Demographic characteristics.

	Total (N = 235)	White matter hyperintensity			P value^†^
		No (N = 21)	Mild (N = 133)	Severe (N = 81)	
Age, y, median [IQR]	63 [54–69]	47 [43–54]	61 [54–66]	69 [63–74]	<0.001
< 65	133 (56.6)	20 (95.2)	89 (66.9)	24 (29.6)	
≥65	102 (43.4)	1 (4.8)	44 (33.1)	57 (70.4)	
Sex, woman	76 (32.3)	6 (28.6)	34 (25.6)	36 (44.4)	0.004
Hypertension	178 (75.7)	13 (61.9)	95 (71.4)	70 (86.4)	0.006
Diabetes mellitus	72 (30.6)	8 (38.1)	37 (27.8)	27 (33.3)	0.52
Hyperlipidemia	13 (5.5)	0 (0)	6 (4.5)	7 (8.6)	0.13
Smoking	118 (50.2)	12 (57.1)	79 (59.4)	27 (33.3)	<0.001
NIHSS, median [IQR]	3 [1–6]	2 [1–5]	3 [1–6]	3 [1–7]	0.44
Mild (0–5)	172 (73.2)	16 (76.2)	98 (73.7)	58 (71.6)	
Moderate (6–14)	56 (23.8)	4 (19.0)	33 (24.8)	19 (23.5)	
Severe (≥15)	7 (3.0)	1 (4.8)	2 (1.5)	4 (4.9)	
Cerebral artery atherosclerosis	130 (55.3)	7 (33.3)	60 (45.1)	63 (77.8)	<0.001
None	105 (44.7)	14 (66.7)	73 (54.9)	18 (22.2)	
Intracranial	61 (26.0)	4 (19.0)	28 (21.1)	29 (35.8)	0.013
Extracranial	23 (9.8)	3 (14.3)	15 (11.3)	5 (6.2)	0.18
Combined	46 (19.6)	0 (0)	17 (12.8)	29 (35.8)	<0.001

IQR indicates interquartile range; NIHSS, NIH Stroke Scale

Values are n (%)

^†^ Comparison between severe WMH and others.

### Functional outcomes at 3 months

**Fig 2:**
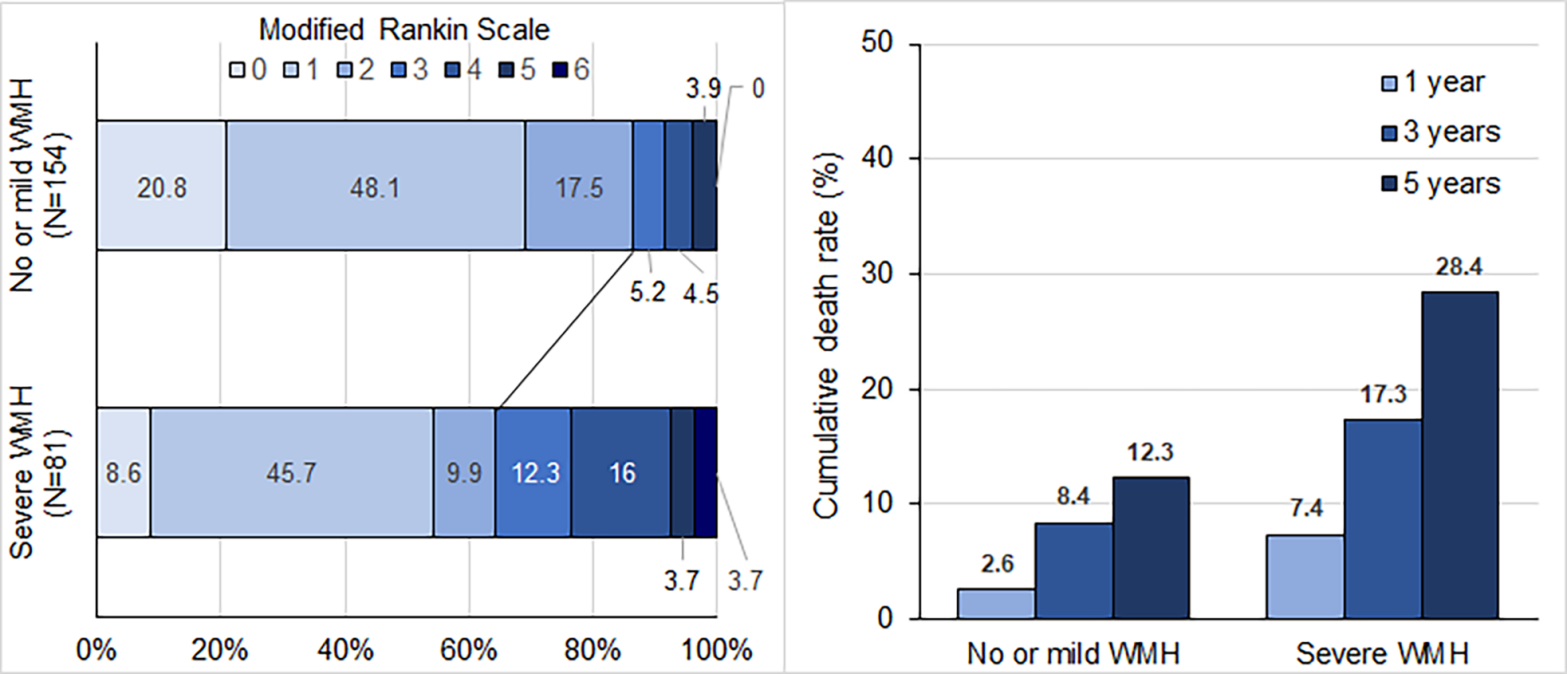
Functional outcomes at 3 months and cumulative death rate. WMH indicates white matter hyperintensities; mRS, modified Rankin Scale.

Poor functional outcomes at 3 months were more common in those with severe WMH (Fig 2). In multivariable analysis, initial NIHSS and severe WMH were independent predictors of poor functional outcome at 3 months. CS patients with severe WMH were 5.25 times (95% CI, 2.07–13.31) more likely to have poor outcomes at 3 months compared to those without (Table 2). The interaction term indicated that age was not a significant modifier of the relationship between short-term functional outcome and WMH severity (P = 0.209).

**Table 2:** Logistic regression analysis for poor outcome at 3 months (mRS score 3–6).

	Unadjusted OR (95% CI)	P value	Adjusted OR (95% CI)	P value
Age^†^	1.01 (0.98–1.04)	0.636	0.98 (0.94–1.02)	0.29
Woman	1.91 (1.00–3.62)	0.05	1.36 (0.59–3.15)	0.47
Hypertension	1.02 (0.49–2.11)	0.96		
Diabetes mellitus	1.53 (0.79–2.94)	0.20		
Hyperlipidemia	2.46 (0.77–7.88)	0.13		
Smoking	0.66 (0.35–1.24)	0.19		
Initial NIHSS^†^	1.39 (1.27–1.53)	<0.001	1.40 (1.27–1.55)	<0.001
Cerebral arteryatherosclerosis				
None	1 (Reference)	NA		
Intracranial	1.30 (0.61–2.77)	0.49		
Extracranial	0.84 (0.26–2.74)	0.78		
Combined	1.11 (0.48–2.60)	0.81		
Severe WMH	3.53 (1.85–6.74)	<0.001	5.25 (2.07–13.31)	<0.001

mRS indicates modified Rankin Scale; OR, odd ratio; NIHSS, NIH Stroke Scale; NA, not applicable; WMH, white matter hyperintensity

^†^ Continuous variables

### Cumulative death rates

Study patients were followed-up for a median of 7.7 years (IQR, 6.7–9.0). During the follow-up period, 64 (27.2%) patients died. The cumulative death rates were 4.3% within 1 year, 11.5% within 3 years, and 17.9% within 5 years. Cumulative death rates were higher in patients with severe WMH than in those with no or mild WMH (7.4% vs. 2.6% within 1 year, 17.3% vs. 8.4% within 3 years, 28.4% vs. 12.3% within 5 years) (Fig 2).

### Univariable and multivariable analyses of long-term mortality

**Fig 3:**
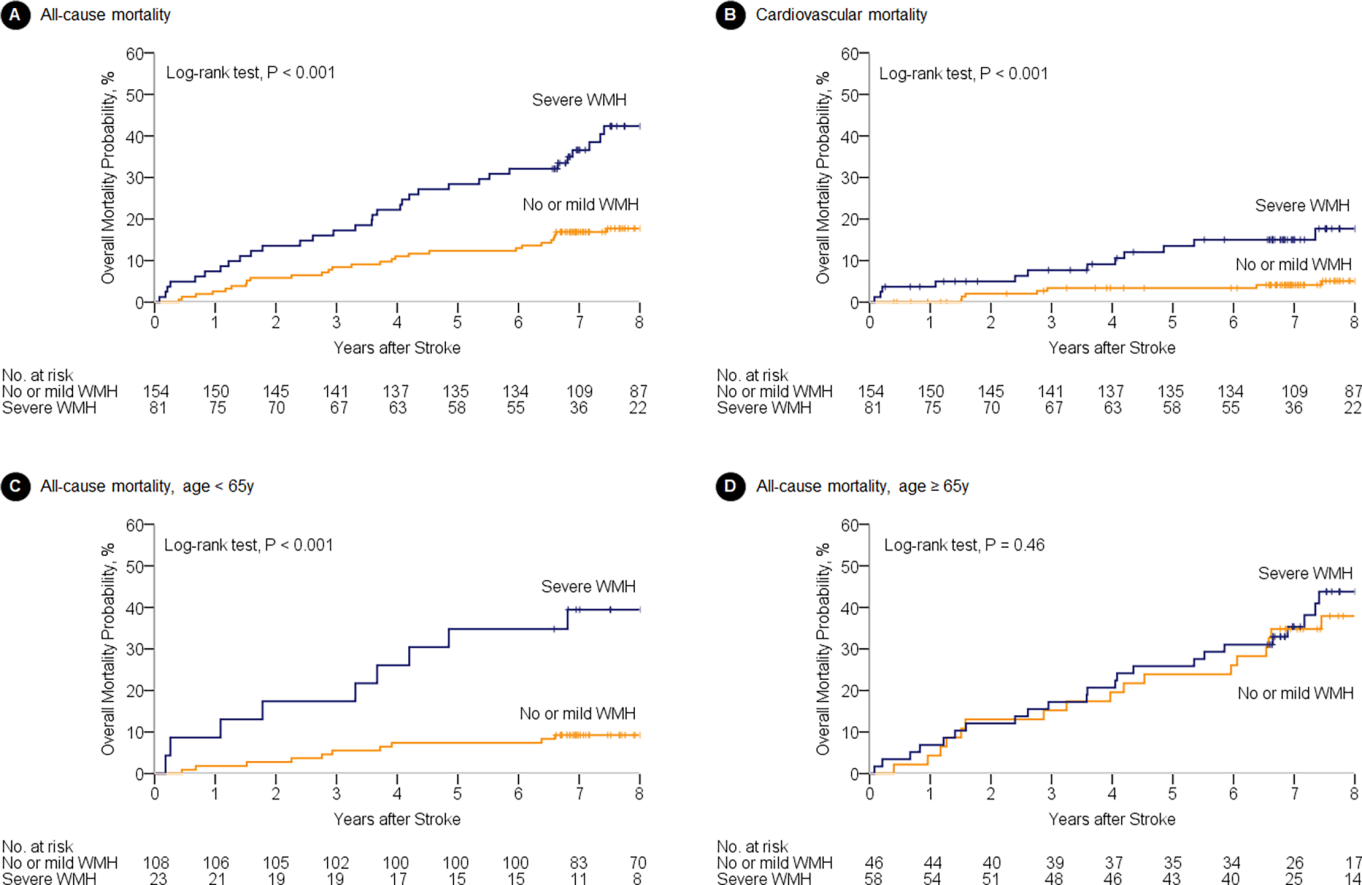
Kaplan-Meier survival analysis. The small vertical ticks on the curves indicate censored patients. WMH indicates white matter hyperintensities.

Kaplan-Meier survival analysis revealed that all-cause mortality and cardiovascular mortality were higher in patients with severe WMH compared to those with no or mild WMH (Fig 3). In Cox regression analyses, old age, woman, and initial stroke severity were independent predictors of long-term mortality in CS patients. CS patients with severe WMH showed a tendency toward a higher mortality (HR 1.72, P = 0.06). Rather, there was a significant interaction between age and the severity of WMH on long-term mortality, whereby the impact of WMH on survival was moderated by age with a negative coefficient (unstandardized beta = -0.057, SE = 0.029, P for interaction = 0.036 for long-term mortality). It means that impact of WMH on long-term mortality was influenced by age. (Table 3).

**Table 3:** Multivariable analysis for survival predictors.

	Unadjusted HR (95% CI)	P value	Adjusted HR (95% CI)	P value	Adjusted HR for Interaction model (95% CI)	P value
Age^†^	1.07 (1.04–1.10)	<0.001	1.07 (1.04–1.10)	<0.001	1.10 (1.06–1.14)	<0.001
Woman	0.85 (0.50–1.46)	0.56	0.41 (0.23–0.73)	0.003	0.42 (0.23–0.75)	0.003
Hypertension	1.39 (0.74–2.60)	0.31				
Diabetes mellitus	1.86 (1.13–3.06)	0.01	1.61 (0.96–2.68)	0.07	1.56(0.92–2.62)	0.10
Hyperlipidemia	1.11 (0.40–3.05)	0.84				
Smoking	0.67 (0.41–1.11)	0.12				
Initial NIHSS^†^	1.09 (1.03–1.14)	0.001	1.12 (1.06–1.19)	<0.001	1.12 (1.05–1.18)	<0.001
Cerebral artery atherosclerosis						
None	1 (Reference)	NA	1 (Reference)	NA	1 (Reference)	NA
Intracranial	1.26 (0.69–2.32)	0.45	0.87 (0.46–1.65)	0.67	0.89 (0.47–1.69)	0.72
Extracranial	0.53 (1.60–1.75)	0.30	0.31 (0.09–1.08)	0.07	0.31 (0.09–1.11)	0.07
Combined	1.87 (1.02–3.44)	0.04	1.01 (0.52–1.99)	0.97	1.02 (0.52–2.01)	0.95
Severe WMH	2.84 (1.73–4.67)	<0.001	1.72 (0.97–3.04)	0.06	79.82 (1.76–3627.97)	0.02
Age*severe WMH					0.94 (0.89–1.00)	0.047

HR indicates hazard ratio; NIHSS, NIH Stroke Scale; NA, not applicable; WMH, white matter hyperintensity

^†^ Continuous variable

### Subgroup analysis for long-term mortality according to age

We further investigated the impact of WMH on long-term mortality in CS patients according to age. Severe WMH was more common in older patients (≥65 years) than younger patients (<65 years) (55.9% vs. 18.0%). Kaplan-Meier survival analysis of younger patients showed that more patients with severe WMH died during follow-up compared to those with no or mild WMH (p<0.001). In contrast, there was no difference in survival rate in older patients with severe WMH (p = 0.46) (Fig 3). Among younger patients, Cox regression analysis revealed that initial stroke severity, diabetes mellitus, and the presence of severe WMH were independent predictors of long-term mortality (HR 3.11, 95% CI, 1.29–7.50). In contrast, there was no association between severe WMH and long-term mortality among older patients (HR 1.19, 95% CI, 0.63–2.23) (Table 4). Cox regression survival analysis revealed that the severity of WMH was independent predictors of cardiovascular in younger CS patients, but not in older CS patients (described in S3 Table).

**Table 4:** Multivariable analysis for survival predictors according to age.

	Young age patients (Age < 65 y)				Old age patients (Age ≥ 65 y)			
	Unadjusted HR (95% CI)	P value	Adjusted HR (95% CI)	P value	Unadjusted HR (95% CI)	P value	Adjusted HR (95% CI)	P value
Age^†^	1.09 (1.01–1.17)	0.02	1.07 (0.99–1.16)	0.08	1.07 (1.02–1.13)	0.01	1.08 (1.02–1.14)	0.01
Woman	0.43 (0.13–1.46)	0.18	0.44 (0.13–1.53)	0.20	0.84 (0.45–1.56)	0.57	0.54 (0.27–1.06)	0.07
Hypertension	1.04 (0.41–2.66)	0.93			1.40 (0.59–3.32)	0.45		
Diabetes mellitus	2.57 (1.11–5.93)	0.03	2.71 (1.09–6.74)	0.03	1.56 (8.36–2.91)	0.16		
Hyperlipidemia	1.95 (0.45–8.33)	0.37			0.68 (0.16–2.80)	0.59		
Smoking	0.69 (0.30–1.60)	0.39			0.87 (0.47–1.63)	0.67		
NIHSS score^†^	1.11 (1.04–1.20)	0.004	1.11 (1.03–1.19)	0.005	1.09 (1.01–1.17)	0.02	1.10 (1.02–1.19)	0.01
Cerebral arteryatherosclerosis								
None	1 (Reference)	NA			1 (Reference)	NA		
Intracranial	1.14 (0.43–3.03)	0.80			1.09 (0.50–2.41)	0.82		
Extracranial	0.34 (0.04–2.58)	0.29			0.85 (0.19–3.76)	0.83		
Combined	1.13 (0.32–4.01)	0.85			1.55 (0.73–3.28)	0.25		
Severe WMH	4.98 (2.14–11.58)	<0.001	3.11 (1.29–7.50)	0.01	1.26 (0.68–2.34)	0.46	1.19 (0.63–2.23)	0.60

HR indicates hazard ratio; NIHSS, NIH Stroke Scale; NA, not applicable; WMH, white matter hyperintensity

^†^ Continuous variable

## Discussion

In this study, we demonstrated that 1) severe WMH was substantial in CS patients, 2) accompanying severe WMH was independently associated with short-term outcome in CS patients, and 3) the impacts of white matter hyperintensities on long-term outcomes in CS patients were different according to age. Younger CS patients with severe WMH had higher death rates compared to those with no or mild WMH, but the same distinction was not seen in older patients. Because WMH is a chronic process and CS is frequent even in the young patients, major strength of our study is that we conducted long-term follow-up over 8 years in a large sample of CS patients.

We found that severe WMH was present in 34.5% of CS patients. Older patients more frequently had severe WMH (55.9%). This finding is in line with those of previous reports that identified old age as a strong predictor of WMH [27]. Of note, substantial proportion of younger CS patients also had severe WMH (18.0%). The detection of WMH has been increasing in the general population as MRI has become more popular. The reported prevalence of WMH in stroke patients varies among studies, from 6.8% to 44.4% [28, 29]. This variability may reflect differences in demographic characteristics among study populations including age, race, ethnicity, and imaging protocols. Our study showed that severe WMH were frequently found even in younger CS patients. Recent large national study also confirmed that age and hypertension is major determinant of WMH degree [30].

We presumed the mechanisms of why severe WMH in CS patients with young age was associated with higher long-term mortality. First, because the mechanism of CS is unknown and heterogeneous, some CS patients may have atherosclerotic stenosis or paroxysmal atrial fibrillation, which may bring poor long-term prognosis [2]. In our data, the presence of less than 50% cerebral artery atherosclerosis, especially in the intracranial artery, was correlated with severity of WMH. We previously reported that aortic atheroma burden was closely associated with high degrees of WMH [23]. Considering previous reports of sharing vascular risk factors between large artery atherosclerosis and WMH [24, 31], severe WMH may be a surrogate marker of atherosclerotic burden. High atherosclerotic burden in multiple vascular beds is known to be associated with poor prognosis in stroke patients [32]. Second, the presence of severe WMH may imply hidden cardioembolic sources. Severe WMH is associated with atrial fibrillation, patent foramen ovale, or atrial septal aneurysm [33, 34]. Long-term monitoring of the heart rhythm can identify paroxysmal atrial fibrillation in up to 16% of patients with CS [35]. Considering the patients with cardioembolism have the worst outcomes, it may substantially increase the risk for stroke recurrence and long-term mortality. Third, we demonstrated that severe WMH was an independent predictor of short-term outcomes in CS patients. Because poor short-term prognosis is a major determinant of long-term outcome [36]. poor short-term outcomes in CS patients with severe WMH may lead to higher long-term mortality. Fourth, as younger patients are less likely to have comorbidities other than stroke, higher WMH burden may suggest brain vulnerabilities in younger CS patients. Our study revealed that the severity of WMH was independent predictors of cardiovascular mortality in younger CS patients, but not of non-cardiovascular mortality. In line with our findings, recent reports suggest the presence of severe WMH in young stroke patients may indicate accelerated cerebral aging, which may in turn indicate increased vulnerability to vascular risk factors [37]. In contrast, our study showed that older patients had similar cardiovascular and non-cardiovascular mortality rates whether they had severe WMH or not. Since elderly people are likely to die with multiple comorbidities, the association between severe WMH and long-term mortality rates may be diluted. Other possible explanations include higher stroke recurrence rates, poor drug compliance, and lower motivation levels regarding life style modifications in elderly patients. Taken together, the finding of this study suggested that WMH burden may be an appropriate surrogate imaging marker to determine long-term outcome especially in younger CS patients. To confirm the exact mechanisms of long-term outcomes in CS patients with WMH, a prospective long-term follow-up study is needed.

We found that stroke recovery at 3 months was poor in CS patients with severe WMH. The mechanisms underlying the influence of WMH on short-term functional outcomes are also needed to define. There are several hypotheses. First, patients with severe WMH were 1.5 times more likely to have stroke recurrence within 90 days [29]. Symptomatic or asymptomatic stroke recurrence is highly associated with short-term functional recovery. Second, WMH regions have reduced vascular density and cerebral blood flow, which may lead to infarct growth after ischemic events during the acute period [38]. Third, the presence of dysfunctional neuronal connectivity in patients with WMH can lead to poor recovery after stroke. Neuropathological studies showed decreased neuronal connectivity in WMH areas where pathological findings included demyelination, loss of axons and oligodendrocytes, and astrocytic gliosis in white matter [39]. This may result in decreased functional connectivity between distant cortical regions [40]. Taken together, these lines of evidence indicate that severe WMH may impair plasticity and recovery after ischemic stroke. Fourth, because WMH is closely associated with cognitive dysfunction, post stroke depression, decreased motivation, and poor cooperation during rehabilitation, it may also affect stroke outcomes [8, 21, 41, 42].

Our study has several limitations. First, there is a possibility of selection bias due to the retrospective design. Therefore, we enrolled consecutive ischemic stroke patients and conducted long-term follow-up. Depth of cardiac evaluation and presence of hidden cardioembolic source may influence the results. Second, 76.3% of stroke had NIHSS 0–5. Although NIHSS is a major predictor of poor stroke outcome, the stroke severity is usually mild in CS patients than other stroke subtypes. Also, there were only 4 deaths within 30 days in our study. This poses the possibility that stroke patients with early mortality who were CS patients might have been excluded because they had no chance of etiology evaluation such as cardiac evaluation and brain MRI. Therefore, further large study is needed to include severe stroke patients. Third, young patients with extensive WMH could also have another underlying pathophysiology of WMH, for example hereditary SVD [8]. We didn’t consider such etiologies of WMH and it might have influenced our results. Fourth, our patient cohort was entirely of East Asian descent. Studies for different ethnicities are needed.

## Conclusions

We demonstrated that severe WMH in CS patients is associated with poor short-term functional outcomes. The impacts of WMH on long-term outcomes in CS patients with WMH differed according to age. Younger CS patients with severe WMH had higher death rates compared to those with no or mild WMH, whereas the same distinction was not found in older patients. Taken together, WMH grading might be an age specific prognostic marker in CS patients.

## Supporting information

S1 Table Characteristics of excluded patients. NIHSS indicates NIH stroke scale; mRS, modified Rankin Scale.
(DOCX)

S2 Table Logistic regression analysis for factors associated with severe WMH. WMH indicates white matter hyperintensities.
(DOCX)

S3 Table Subgroup analysis according to age group for the impact of white matter hyperintensity on cardiovascular and non-cardiovascular mortality in cryptogenic stroke patients. WMH indicates white matter hyperintensity; CV, cardiovascular.
^†^Adjusted for age, sex, hypertension, diabetes mellitus and NIH stroke scale.
(DOCX)

S4 Table Etiologic evaluations according to degree of WMH. WMH indicates white matter hyperintensities; CTA, CT angiography; MRA, MR angiography; DSA, digital subtraction angiography; TEE, transesophageal echocardiography; TTE, transthoracic echocardiography.
Values are n (%).
(DOCX)

S1 File Data set of all patients who were included in the final cohort. MI indicates myocardial infarction; CV, cardiovascular; NIHSS, NIH stroke scale; mRS, modified Rankin Scale; TEE, Transesophageal echocardiography; TTE, Transthoracic echocardiography; WMH, white matter hyperintensities; PWMH, periventricular white matter hyperintensities; DWMH, deep white matter hyperintensities.
(XLSX)

## References

[1] Li L, Yiin GS, Geraghty OC, Schulz UG, Kuker W, Mehta Z, et al Incidence, outcome, risk factors, and long-term prognosis of cryptogenic transient ischaemic attack and ischaemic stroke: a population-based study. The Lancet Neurology. 2015;14(9):903–13. Epub 2015/08/01. doi: 10.1016/S1474-4422(15)00132-5 . 26227434 10.1016/S1474-4422(15)00132-5 PMC5714616

[2] Petty GW, Brown RD Jr., Whisnant JP, Sicks JD, O'Fallon WM, Wiebers DO. Ischemic stroke subtypes: a population-based study of functional outcome, survival, and recurrence. Stroke; a journal of cerebral circulation. 2000;31(5):1062–8. Epub 2000/05/08. . 10797166 10.1161/01.str.31.5.1062

[3] Kolominsky-Rabas PL, Weber M, Gefeller O, Neundoerfer B, Heuschmann PU. Epidemiology of ischemic stroke subtypes according to TOAST criteria: incidence, recurrence, and long-term survival in ischemic stroke subtypes: a population-based study. Stroke; a journal of cerebral circulation. 2001;32(12):2735–40. Epub 2001/12/12. . 11739965 10.1161/hs1201.100209

[4] Adams HP Jr., Davis PH, Leira EC, Chang KC, Bendixen BH, Clarke WR, et al Baseline NIH Stroke Scale score strongly predicts outcome after stroke: A report of the Trial of Org 10172 in Acute Stroke Treatment (TOAST). Neurology. 1999;53(1):126–31. Epub 1999/07/17. . 10408548 10.1212/wnl.53.1.126

[5] Uchino K, Billheimer D, Cramer SC. Entry criteria and baseline characteristics predict outcome in acute stroke trials. Stroke; a journal of cerebral circulation. 2001;32(4):909–16. 10.1161/01.str.32.4.909 11283391

[6] Pantano P, Caramia F, Bozzao L, Dieler C, von Kummer R. Delayed increase in infarct volume after cerebral ischemia. Stroke; a journal of cerebral circulation. 1999;30(3):502–7. 10.1161/01.str.30.3.502 10066843

[7] Tveiten A, Ljostad U, Mygland A, Naess H. Leukoaraiosis is associated with short- and long-term mortality in patients with intracerebral hemorrhage. Journal of stroke and cerebrovascular diseases: the official journal of National Stroke Association. 2013;22(7):919–25. Epub 2013/02/26. doi: 10.1016/j.jstrokecerebrovasdis.2013.01.017 . 23433781 10.1016/j.jstrokecerebrovasdis.2013.01.017

[8] Pantoni L. Cerebral small vessel disease: from pathogenesis and clinical characteristics to therapeutic challenges. The Lancet Neurology. 2010;9(7):689–701. Epub 2010/07/09. doi: 10.1016/S1474-4422(10)70104-6 . 20610345 10.1016/S1474-4422(10)70104-6

[9] Zhang J, Puri AS, Khan MA, Goddeau RP Jr., Henninger N. Leukoaraiosis predicts a poor 90-day outcome after endovascular stroke therapy. AJNR American journal of neuroradiology. 2014;35(11):2070–5. Epub 2014/07/06. doi: 10.3174/ajnr.A4029 . 24994827 10.3174/ajnr.A4029 PMC7965186

[10] Debette S, Markus HS. The clinical importance of white matter hyperintensities on brain magnetic resonance imaging: systematic review and meta-analysis. BMJ (Clinical research ed). 2010;341:c3666 Epub 2010/07/28. doi: 10.1136/bmj.c3666 ; PubMed Central PMCID: PMCPmc2910261. 20660506 10.1136/bmj.c3666 PMC2910261

[11] van der Holst HM, van Uden IW, Tuladhar AM, de Laat KF, van Norden AG, Norris DG, et al Factors Associated With 8-Year Mortality in Older Patients With Cerebral Small Vessel Disease: The Radboud University Nijmegen Diffusion Tensor and Magnetic Resonance Cohort (RUN DMC) Study. JAMA neurology. 2016;73(4):402–9. Epub 2016/02/03. doi: 10.1001/jamaneurol.2015.4560 . 26831360 10.1001/jamaneurol.2015.4560

[12] Koton S, Schwammenthal Y, Merzeliak O, Philips T, Tsabari R, Orion D, et al Cerebral leukoaraiosis in patients with stroke or TIA: clinical correlates and 1-year outcome. European journal of neurology. 2009;16(2):218–25. Epub 2009/01/14. doi: 10.1111/j.1468-1331.2008.02389.x . 19138336 10.1111/j.1468-1331.2008.02389.x

[13] Arsava EM, Rahman R, Rosand J, Lu J, Smith EE, Rost NS, et al Severity of leukoaraiosis correlates with clinical outcome after ischemic stroke. Neurology. 2009;72(16):1403–10. Epub 2009/04/22. doi: 10.1212/WNL.0b013e3181a18823 ; PubMed Central PMCID: PMCPmc2677507. 19380699 10.1212/WNL.0b013e3181a18823 PMC2677507

[14] Miyao S, Takano A, Teramoto J, Takahashi A. Leukoaraiosis in relation to prognosis for patients with lacunar infarction. Stroke; a journal of cerebral circulation. 1992;23(10):1434–8. Epub 1992/10/01. . 1412580 10.1161/01.str.23.10.1434

[15] Henninger N, Lin E, Baker SP, Wakhloo AK, Takhtani D, Moonis M. Leukoaraiosis predicts poor 90-day outcome after acute large cerebral artery occlusion. Cerebrovascular diseases (Basel, Switzerland). 2012;33(6):525–31. Epub 2012/04/28. doi: 10.1159/000337335 . 22538962 10.1159/000337335

[16] Lee BI, Nam HS, Heo JH, Kim DI. Yonsei Stroke Registry. Analysis of 1,000 patients with acute cerebral infarctions. Cerebrovascular diseases (Basel, Switzerland). 2001;12(3):145–51. Epub 2001/10/20. doi: 47697. doi: 10.1159/000047697 . 11641577 10.1159/000047697

[17] Cho HJ, Choi HY, Kim YD, Nam HS, Han SW, Ha JW, et al Transoesophageal echocardiography in patients with acute stroke with sinus rhythm and no cardiac disease history. Journal of neurology, neurosurgery, and psychiatry. 2010;81(4):412–5. Epub 2009/12/08. doi: 10.1136/jnnp.2009.190322 . 19965855 10.1136/jnnp.2009.190322

[18] Yoo J, Yang JH, Choi BW, Kim YD, Nam HS, Choi HY, et al The frequency and risk of preclinical coronary artery disease detected using multichannel cardiac computed tomography in patients with ischemic stroke. Cerebrovascular diseases (Basel, Switzerland). 2012;33(3):286–94. Epub 2012/01/31. doi: 10.1159/000334980 . 22286013 10.1159/000334980

[19] Song TJ, Kim J, Lee HS, Nam CM, Nam HS, Kim YD, et al Distribution of cerebral microbleeds determines their association with impaired kidney function. Journal of clinical neurology (Seoul, Korea). 2014;10(3):222–8. Epub 2014/07/22. doi: 10.3988/jcn.2014.10.3.222 ; PubMed Central PMCID: PMCPmc4101099. 25045375 10.3988/jcn.2014.10.3.222 PMC4101099

[20] Adams HP Jr., Bendixen BH, Kappelle LJ, Biller J, Love BB, Gordon DL, et al Classification of subtype of acute ischemic stroke. Definitions for use in a multicenter clinical trial. TOAST. Trial of Org 10172 in Acute Stroke Treatment. Stroke; a journal of cerebral circulation. 1993;24(1):35–41. Epub 1993/01/01. . 7678184 10.1161/01.str.24.1.35

[21] Wardlaw JM, Smith EE, Biessels GJ, Cordonnier C, Fazekas F, Frayne R, et al Neuroimaging standards for research into small vessel disease and its contribution to ageing and neurodegeneration. The Lancet Neurology. 2013;12(8):822–38. Epub 2013/07/23. doi: 10.1016/S1474-4422(13)70124-8 ; PubMed Central PMCID: PMCPmc3714437. 23867200 10.1016/S1474-4422(13)70124-8 PMC3714437

[22] Fazekas F, Chawluk JB, Alavi A, Hurtig HI, Zimmerman RA. MR signal abnormalities at 1.5 T in Alzheimer's dementia and normal aging. AJR American journal of roentgenology. 1987;149(2):351–6. Epub 1987/08/01. doi: 10.2214/ajr.149.2.351 . 3496763 10.2214/ajr.149.2.351

[23] Song TJ, Kim YD, Yoo J, Kim J, Chang HJ, Hong GR, et al Association between Aortic Atheroma and Cerebral Small Vessel Disease in Patients with Ischemic Stroke. Journal of stroke. 2016 Epub 2016/08/05. doi: 10.5853/jos.2016.00171 . 27488980 10.5853/jos.2016.00171 PMC5066433

[24] Lee SJ, Kim JS, Chung SW, Kim BS, Ahn KJ, Lee KS. White matter hyperintensities (WMH) are associated with intracranial atherosclerosis rather than extracranial atherosclerosis. Archives of gerontology and geriatrics. 2011;53(2):e129–32. Epub 2010/08/31. doi: 10.1016/j.archger.2010.07.008 . 20800910 10.1016/j.archger.2010.07.008

[25] Nam HS, Kim HC, Kim YD, Lee HS, Kim J, Lee DH, et al Long-term mortality in patients with stroke of undetermined etiology. Stroke; a journal of cerebral circulation. 2012;43(11):2948–56. Epub 2012/08/31. doi: 10.1161/strokeaha.112.661074 . 22933583 10.1161/STROKEAHA.112.661074

[26] Govan L, Langhorne P, Weir CJ. Categorizing stroke prognosis using different stroke scales. Stroke; a journal of cerebral circulation. 2009;40(10):3396–9. Epub 2009/08/08. doi: 10.1161/strokeaha.109.557645 . 19661471 10.1161/STROKEAHA.109.557645

[27] Longstreth WT Jr., Manolio TA, Arnold A, Burke GL, Bryan N, Jungreis CA, et al Clinical correlates of white matter findings on cranial magnetic resonance imaging of 3301 elderly people. The Cardiovascular Health Study. Stroke; a journal of cerebral circulation. 1996;27(8):1274–82. Epub 1996/08/01. . 8711786 10.1161/01.str.27.8.1274

[28] Wiszniewska M, Devuyst G, Bogousslavsky J, Ghika J, van Melle G. What is the significance of leukoaraiosis in patients with acute ischemic stroke? Archives of neurology. 2000;57(7):967–73. Epub 2000/07/13. . 10891978 10.1001/archneur.57.7.967

[29] Kim GM, Park KY, Avery R, Helenius J, Rost N, Rosand J, et al Extensive leukoaraiosis is associated with high early risk of recurrence after ischemic stroke. Stroke; a journal of cerebral circulation. 2014;45(2):479–85. Epub 2013/12/29. doi: 10.1161/strokeaha.113.003004 . 24370756 10.1161/STROKEAHA.113.003004

[30] Ryu W-S, Woo S-H, Schellingerhout D, Chung MK, Kim CK, Jang MU, et al Grading and interpretation of white matter hyperintensities using statistical maps. Stroke; a journal of cerebral circulation. 2014;45(12):3567–75. 10.1161/STROKEAHA.114.006662 25388424

[31] Lee SJ, Kim JS, Lee KS, An JY, Kim W, Kim YI, et al The leukoaraiosis is more prevalent in the large artery atherosclerosis stroke subtype among Korean patients with ischemic stroke. BMC neurology. 2008;8:31 Epub 2008/08/09. doi: 10.1186/1471-2377-8-31 ; PubMed Central PMCID: PMCPmc2532686. 18687146 10.1186/1471-2377-8-31 PMC2532686

[32] Gerdes VE, Kwa VI, ten Cate H, Brandjes DP, Buller HR, Stam J. Cerebral white matter lesions predict both ischemic strokes and myocardial infarctions in patients with established atherosclerotic disease. Atherosclerosis. 2006;186(1):166–72. Epub 2005/08/16. doi: 10.1016/j.atherosclerosis.2005.07.008 . 16098981 10.1016/j.atherosclerosis.2005.07.008

[33] de Leeuw FE, de Groot JC, Oudkerk M, Kors JA, Hofman A, van Gijn J, et al Atrial fibrillation and the risk of cerebral white matter lesions. Neurology. 2000;54(9):1795–801. Epub 2000/05/10. . 10802786 10.1212/wnl.54.9.1795

[34] Ueno Y, Shimada Y, Tanaka R, Miyamoto N, Tanaka Y, Hattori N, et al Patent Foramen Ovale with Atrial Septal Aneurysm May Contribute to White Matter Lesions in Stroke Patients. Cerebrovascular Diseases. 2010;30(1):15–22. doi: 10.1159/000313439 20424440 10.1159/000313439

[35] Sanna T, Diener HC, Passman RS, Di Lazzaro V, Bernstein RA, Morillo CA, et al Cryptogenic stroke and underlying atrial fibrillation. The New England journal of medicine. 2014;370(26):2478–86. Epub 2014/06/26. doi: 10.1056/NEJMoa1313600 . 24963567 10.1056/NEJMoa1313600

[36] Breteler MM, van Swieten JC, Bots ML, Grobbee DE, Claus JJ, van den Hout JH, et al Cerebral white matter lesions, vascular risk factors, and cognitive function in a population-based study: the Rotterdam Study. Neurology. 1994;44(7):1246–52. Epub 1994/07/01. . 8035924 10.1212/wnl.44.7.1246

[37] Arntz RM, van den Broek SM, van Uden IW, Ghafoorian M, Platel B, Rutten-Jacobs LC, et al Accelerated development of cerebral small vessel disease in young stroke patients. Neurology. 2016;87(12):1212–9. Epub 2016/08/16. doi: 10.1212/WNL.0000000000003123 . 27521431 10.1212/WNL.0000000000003123 PMC5035980

[38] O'Sullivan M, Lythgoe DJ, Pereira AC, Summers PE, Jarosz JM, Williams SC, et al Patterns of cerebral blood flow reduction in patients with ischemic leukoaraiosis. Neurology. 2002;59(3):321–6. Epub 2002/08/15. . 12177363 10.1212/wnl.59.3.321

[39] Yamanouchi H, Sugiura S, Tomonaga M. Decrease in nerve fibres in cerebral white matter in progressive subcortical vascular encephalopathy of Binswanger type. An electron microscopic study. Journal of neurology. 1989;236(7):382–7. Epub 1989/10/01. . 2809638 10.1007/BF00314894

[40] Nordahl CW, Ranganath C, Yonelinas AP, Decarli C, Fletcher E, Jagust WJ. White matter changes compromise prefrontal cortex function in healthy elderly individuals. Journal of cognitive neuroscience. 2006;18(3):418–29. Epub 2006/03/04. doi: 10.1162/089892906775990552 ; PubMed Central PMCID: PMCPmc3776596. 16513006 10.1162/089892906775990552 PMC3776596

[41] Firbank MJ, Teodorczuk A, van der Flier WM, Gouw AA, Wallin A, Erkinjuntti T, et al Relationship between progression of brain white matter changes and late-life depression: 3-year results from the LADIS study. The British journal of psychiatry: the journal of mental science. 2012;201(1):40–5. Epub 2012/05/26. doi: 10.1192/bjp.bp.111.098897 . 22626634 10.1192/bjp.bp.111.098897

[42] Schaapsmeerders P, Tuladhar AM, Arntz RM, Franssen S, Maaijwee NAM, Rutten-Jacobs LCA, et al Remote Lower White Matter Integrity Increases the Risk of Long-Term Cognitive Impairment After Ischemic Stroke in Young Adults. Stroke; a journal of cerebral circulation. 2016;47(10):2517–25. doi: 10.1161/strokeaha.116.014356 27625378 10.1161/STROKEAHA.116.014356

