# Supplementary material for: Impact of white matter hyperintensities on the prognosis of cryptogenic stroke patients
Source: PLoS One. 2018 Apr 27;13(4):e0196014. doi: 10.1371/journal.pone.0196014 (PMC5922577; doi:10.1371/journal.pone.0196014)
Supplement: S1 Table — NIHSS indicates NIH stroke scale; mRS, modified Rankin Scale. (DOCX) [file pone.0196014.s001.docx]

**S1 Table. Characteristics of excluded patients**

|  | Enrolled patients  (n=235) | Excluded patients  (n=364) | P value |
| --- | --- | --- | --- |
| Age | 63 [54-69] | 66 [57-73] | 0.001 |
| Woman | 76 (32.3%) | 154 (42.3%) | 0.01 |
| Hypertension | 178 (75.7%) | 270 (74.2%) | 0.67 |
| Diabetes mellitus | 72 (30.6%) | 123 (33.8%) | 0.42 |
| Hyperlipidemia | 34 (7.8%) | 19 (11.9%) | 0.11 |
| Smoking | 118 (50.2%) | 156 (42.9%) | 0.08 |
| NIHSS | 3 [1-5] | 3 [1-6] | 0.60 |
| mRS (3-6) at 3 months | 50 (21.3%) | 74 (20.3%) | 0.78 |
| Mortality | 64 (27.2%) | 123 (33.8%) | 0.09 |

NIHSS indicates NIH stroke scale; mRS, modified Rankin Scale
