# Supplementary material for: Impact of white matter hyperintensities on the prognosis of cryptogenic stroke patients
Source: PLoS One. 2018 Apr 27;13(4):e0196014. doi: 10.1371/journal.pone.0196014 (PMC5922577; doi:10.1371/journal.pone.0196014)
Supplement: S2 Table — WMH indicates white matter hyperintensities. (DOCX) [file pone.0196014.s002.docx]

**S2 Table. Logistic regression analysis for factors associated with severe WMH**

|  | Unadjusted OR (95% CI) | P value | Adjusted OR (95% CI) | P value |
| --- | --- | --- | --- | --- |
| Age | 1.11 (1.07-1.15) | <0.001 | 1.09 (1.05-1.32) | <0.001 |
| Woman | 2.28 (1.29-4.02) | 0.004 | 0.80 (0.35-1.83) | 0.59 |
| Hypertension | 2.71 (1.31-5.59) | 0.007 | 2.22 (0.91-5.40) | 0.80 |
| Diabetes mellitus | 1.21 (0.68-2.16) | 0.52 |  |  |
| Smoking | 0.35 (0.20-0.61) | <0.001 | 0.41 (0.18-0.94) | 0.34 |
| Hyperlipidemia | 2.33 (0.76-7.19) | 0.14 |  |  |
| Cerebral artery atherosclerosis |  |  |  |  |
| None | 1 (Reference) | NA | 1 (Reference) | NA |
| Intracranial | 4.38 (2.14-8.95) | <0.001 | 2.65 (1.19-5.92) | 0.02 |
| Extracranial | 1.34 (0.44-4.09) | 0.60 | 1.35 (0.41-4.47) | 0.62 |
| Combined | 8.25 (3.76-18.07) | <0.001 | 4.72 (2.01-11.10) | <0.001 |

WMH indicates white matter hyperintensities
