# Supplementary material for: Impact of white matter hyperintensities on the prognosis of cryptogenic stroke patients
Source: PLoS One. 2018 Apr 27;13(4):e0196014. doi: 10.1371/journal.pone.0196014 (PMC5922577; doi:10.1371/journal.pone.0196014)
Supplement: S3 Table — WMH indicates white matter hyperintensity; CV, cardiovascular. †Adjusted for age, sex, hypertension, diabetes mellitus and NIH stroke scale. (DOCX) [file pone.0196014.s003.docx]

**S3 Table. Subgroup analysis according to age group for the impact of white matter hyperintensity on cardiovascular and non-cardiovascular mortality in cryptogenic stroke patients**

|  |  | Age < 65y | | | | Age ≥ 65y | | | |
| --- | --- | --- | --- | --- | --- | --- | --- | --- | --- |
|  |  | Unadjusted HR (95% CI) | P value | Adjusted HR (95% CI)^†^ | P value | Unadjusted HR (95% CI) | P value | Adjusted HR (95% CI)^†^ | P value |
| CV mortality | No or mild WMH | 1 (Reference) | NA | 1 (Reference) | NA | 1 (Reference) | NA | 1 (Reference) | NA |
|  | Severe WMH | 7.38 (1.97-27.65) | 0.003 | 3.82 (0.98-14.89) | 0.05 | 2.66 (0.72-9.87) | 0.14 | 2.53 (0.64-10.06) | 0.19 |
| Non-CV mortality | No or mild WMH | 1 (Reference) | NA | 1 (Reference) | NA | 1 (Reference) | NA | 1 (Reference) | NA |
|  | Severe WMH | 3.77 (1.23-11.58) | 0.02 | 2.55 (0.78-8.35) | 0.12 | 0.97 (0.47-1.99) | 0.97 | 0.83 (0.40-1.74) | 0.62 |

WMH indicates white matter hyperintensity; CV, cardiovascular

^†^Adjusted for age, sex, hypertension, diabetes mellitus and NIH stroke scale
