# Supplementary material for: Impact of white matter hyperintensities on the prognosis of cryptogenic stroke patients
Source: PLoS One. 2018 Apr 27;13(4):e0196014. doi: 10.1371/journal.pone.0196014 (PMC5922577; doi:10.1371/journal.pone.0196014)
Supplement: S4 Table — WMH indicates white matter hyperintensities; CTA, CT angiography; MRA, MR angiography; DSA, digital subtraction angiography; TEE, transesophageal echocardiography; TTE, transthoracic echocardiography. Values are n (%). (DOCX) [file pone.0196014.s004.docx]

**S4 Table. Etiologic evaluations according to degree of WMH**

|  | Total (N=438) | No or mild WMH (N=274) | Severe WMH (N=164) | P value |
| --- | --- | --- | --- | --- |
| Angiographic evaluations | 438 (100) | 274 (100) | 164 (100) | NS |
| CTA | 95 (21.7) | 61 (22.3) | 34 (20.7) |  |
| MRA | 387 (88.4) | 238 (86.9) | 149 (90.9) |  |
| DSA | 120 (27.4) | 84 (30.7) | 36 (22.0) |  |
| Cardiac image | 285 (65.1) | 182 (66.4) | 103 (62.8) | 0.44 |
| Heart CT | 56 (12.8) | 29 (10.6) | 27 (16.5) |  |
| Echocardiography | 266 (60.7) | 172 (62.8) | 94 (57.3) |  |
| TEE | 246 (56.2) | 166 (60.6) | 80 (48.8) |  |
| TTE | 31 (7.1) | 13 (4.7) | 18 (11.0) |  |
| Continuous EKG monitoring | 388 (88.6) | 246 (89.8) | 142 (86.6) | 0.31 |
|  |  |  |  |  |
| Both cardiac image and EKG monitoring | 235 (53.7) | 154 (56.2) | 81 (49.4) | 0.17 |

WMH indicates white matter hyperintensities; CTA, CT angiography; MRA, MR angiography; DSA, digital subtraction angiography; TEE, transesophageal echocardiography; TTE, transthoracic echocardiography.

Values are n (%).
